# The ISGyP Endocervical Adenocarcinoma Project

**DOI:** 10.1097/PGP.0000000000000759

**Published:** 2021-02-09

**Authors:** Esther Oliva

**Affiliations:** Pathology Department, Massachusetts General Hospital, Boston, MA

It is with great enthusiasm that I am addressing all of you to introduce this special issue of the *International Journal of Gynecologic Pathology* dedicated in its entirety to the ISGyP Endocervical Adenocarcinoma Project.

I would like to thank most deeply the “Endocervical Adenocarcinoma Group” for the tremendous efforts of each of the members in the undertaking and completion of this endeavor which has resulted in nine individual articles related to endocervical adenocarcinoma (highlighted below). I also want to thank all the members of the Society for their participation and support in this project. They helped to provide a set of consensus recommendations relating to many problematic aspects of endocervical adenocarcinoma as a society, recommendations of best-practice guidelines for our daily work. I also want to thank Dr Elvio Silva for his advice and support.

**FIG. 1:**
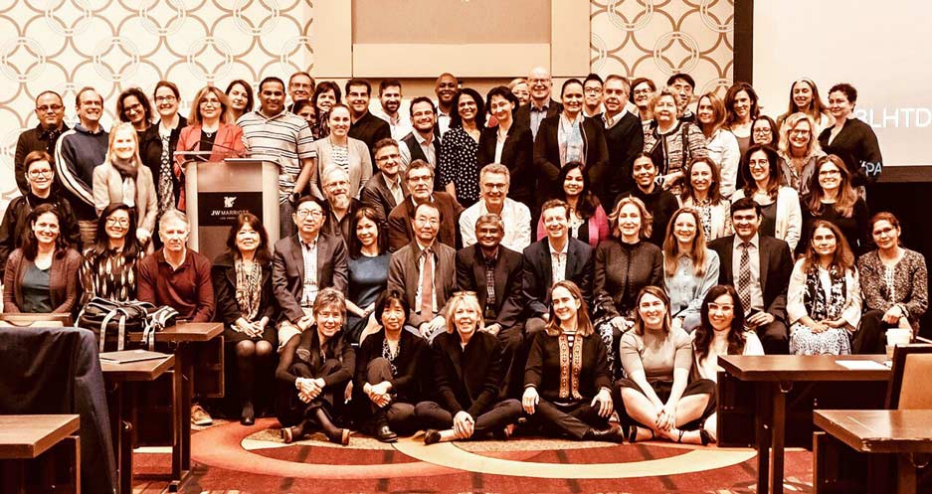
Gynecologic pathologists that participated at the Endocervical Adenocarcinoma Forum Saturday, February 29, Los Angeles.

The ISGyP endocervical adenocarcinoma project was launched in the middle of 2019 to build evidence-based and/or best-practice recommendations for pathologic reporting of all aspects of cervical adenocarcinoma and had 3 main aims: (1) to understand the spectrum of current practices in pathologic evaluation (management, gross, diagnosis, and classification) and reporting of endocervical adenocarcinoma with a review of the literature including controversial areas as well as a member survey; (2) to improve global reproducibility of endocervical adenocarcinoma classification and pathologic reporting; and (3) to assess prognostic significance of WHO/IECC endocervical adenocarcinoma classification and Silva pattern-based classification of human papillomavirus (HPV)-associated adenocarcinomas. A dedicated meeting was held on February 29, 2020, in advance of the annual ISGyP Symposium and USCAP meetings held in Los Angeles (Fig. 1).

The articles that constitute this special issue of the *International Journal of Gynecologic Pathology* include a survey of current individual practices, results of online training and self-assessment in the histopathologic classification of endocervical adenocarcinoma and diagnosis of patterns of invasion, and evidence-based and/or practice-based recommendations on gross examination and processing, including intraoperative evaluation, distinction of in situ from invasive adenocarcinoma and Silva patterns of invasion; grading of endocervical adenocarcinoma; tumor typing including immunohistochemistry; staging of endocervical adenocarcinoma; and predictive markers. A brief report on participation includes results of voting on the recommendations that have been put forward.

As the first step of this vast endocervical project, an exhaustive survey was designed to gather information on existing practices regarding grossing, processing, diagnosing and reporting of cervical adenocarcinoma among the members of the society. The survey was completed by 175 members of the society around the world (page S4).

A key task of our society is to be part of the decision strategies. The work of our society can have a tremendous impact in changing practices in our field and in those of our colleagues. Thus, the ISGyP Endocervical Adenocarcinoma Data Collection Project was created with ISGyP members from 112 centers from around the globe offering to participate in contributing anonymized patient data to assess the prognostic significance of WHO/IECC endocervical adenocarcinoma classification and Silva pattern-based classification of HPV-associated adenocarcinomas with the goal of generating an evidence-basis to change current practices. As a result of this collaborative project, data from over 2500 individual cases have been collected and results of the analysis will follow over coming months.

In order to guide the pathologists participating in the data collection project, and to increase proficiency of all ISGyP members in the IECC/WHO classification and the Silva pattern-based assessment, members of the endocervical project designed training and test sets to improve the diagnostic performance in these 2 particular areas and as a first step tested the interobserver agreement between specialized gynecologic pathologists with the goal to identify areas of suboptimal diagnostic performance that may require future educational efforts (page S14).

As we all know, gross examination and processing, including intraoperative evaluation of any specimen is crucial for correct microscopic assessment of tumors which will impact patient staging, treatment and prognosis. In the last years, there has been a trend to underappreciate the importance of specimen grossing which is the basis of any pathology report. The article on gross examination and processing, including intraoperative evaluation of endocervical adenocarcinoma provides recommended approaches to the specimen types that are encountered in daily practice from fragmented or intact LEEP/LLETZ, cold-knife cone, trachelectomy, hysterectomy, pelvic exenteration, and sentinel lymph node specimens including practical recommendations (page S24).

The article on distinction of adenocarcinoma in situ from invasive adenocarcinoma and Silva patterns of invasion highlights the struggle that pathologists face in separating noninvasive from invasive adenocarcinoma in some instances, especially when dealing with HPV-related adenocarcinomas. However, the relatively new Silva pattern of invasion system may alleviate this problem as all patients with pattern A of invasion (~200 among ~1300 patients) with follow-up to date using this system, had stage I tumors and none recurred. Pattern A of invasion is characterized by well-demarcated rounded glands without high-grade atypia, often with a lobular architecture, without associated desmoplasia or associated inflammatory infiltrate or single cells or detached clusters of tumor cells within the stroma. Minimal cribriform or papillary architecture not filling a 4× field (5 mm in diameter), but not solid growth, is allowed. These patients would not require lymphadenectomy, avoiding important potential subsequent comorbidities as part of their treatment. Corroborating the prognostic utility of the Silva pattern of invasion is one of the goals of the collection data of the endocervical adenocarcinoma project (page S48).

Grading of endocervical adenocarcinoma has always been a mystery as no universally applied, validated system has existed for these tumors. Pathologists tend to grade endocervical carcinomas typically following the FIGO system applied to endometrial endometrioid carcinomas. Most important to keep in mind is that as in the endometrium grading only applies to endometrioid carcinomas, in the endocervix, grading should only be applied to HPV-related endocervical adenocarcinomas, as gastric-type, clear cell and mesonephric carcinomas are considered to be high-grade (page S66).

The article on tumor typing including immunohistochemistry touches upon another breakthrough in the field of endocervical adenocarcinoma, the new HPV-based classification. In the 2014 WHO classification, endocervical adenocarcinomas were classified based on their morphologic features, often times not very reproducible. The new classification links etiopathogenesis to morphology using the presence of luminal mitoses and apoptotic bodies as surrogate markers of an HPV-related tumor. This classification appears to be more informative and more relevant for treatment purposes than the prior one and it has been adopted by the 2020 WHO classification of the female genital tumors. Corroborating these findings is another of the goals of the collection data of the endocervical adenocarcinoma project (page S75).

Correct staging of endocervical adenocarcinoma is crucial for patient treatment and prognosis. However, problematic areas exist in the pathologic staging of these tumors, several related to size issues as well as assessment of extracervical involvement (paracervical/adnexal) and reporting of lymph node involvement. Furthermore, some recent changes have been adopted by FIGO in the staging of cervical carcinomas that have required attention and where members of our society have played a key role (page S92).

The literature on prognostic and predictive markers in cancer is blossoming, however, unfortunately, most studies include all tumor types within one group without specification of results by tumor type creating confusion and potential misinterpretation of results (eg, in cervix squamous cell carcinoma and all types of endocervical adenocarcinoma). It is known that the molecular alterations in different subtypes of endocervical adenocarcinoma differ. The article on predictive markers highlights this issue in the literature published on endocervical adenocarcinoma and provides insights on the potential role of some markers [*ERBB2* (*HER2*) mutations for anti-HER2 treatment and PD-L1 expression and immunotherapy] in these tumors (page S102).

The last article gives a project overview and includes a list of participants. A final step was to elicit the views of participants on the ISGyP recommendations put forward in the articles on different topics discussed in this issue, and the polling results for each recommendation are tabulated therein.

There is still work in the pipeline from the ISGyP Endocervical Adenocarcinoma Data Collection project as we are in the process of collating all the data received from centers around the world. I am aware of the innumerable difficulties that have accompanied us this year (related to the pandemic) that have not allowed many members of the society to participate in this project albeit their utmost enthusiasm and perseverance to do so. I am most appreciative of everyone’s efforts.

